# Retention in HIV Care and Predictors of Attrition from Care among HIV-Infected Adults Receiving Combination Anti-Retroviral Therapy in Addis Ababa

**DOI:** 10.1371/journal.pone.0130649

**Published:** 2015-06-26

**Authors:** Legese A. Mekuria, Jan M. Prins, Alemayehu W. Yalew, Mirjam A. G. Sprangers, Pythia T. Nieuwkerk

**Affiliations:** 1 Department of Medical Psychology, Academic Medical Center, University of Amsterdam, Amsterdam, The Netherlands; 2 Department of Epidemiology, Netherlands Institute for Health Sciences/Erasmus University Medical Center, Rotterdam, The Netherlands; 3 School of Public Health, Addis Ababa University, Addis Ababa, Ethiopia; 4 Department of Internal Medicine, Division of Infectious Diseases, Trop Med & AIDS, Academic Medical Center, University of Amsterdam, Amsterdam, The Netherlands; University of Liverpool, UNITED KINGDOM

## Abstract

**Background:**

Patient retention in chronic HIV care is a major challenge following the rapid expansion of combination antiretroviral therapy (cART) in Ethiopia.

**Objective:**

To describe the proportion of patients who are retained in HIV care and characterize predictors of attrition among HIV-infected adults receiving cART in Addis Ababa.

**Method:**

A retrospective analysis was conducted among 836 treatment naïve patients, who started cART between May 2009 and April 2012. Patients were randomly selected from ten health-care facilities, and their current status in HIV care was determined based on routinely available data in the medical records. Patients lost to follow-up (LTFU) were traced by telephone. Kaplan-Meier technique was used to estimate survival probabilities of retention and Cox proportional hazards regression was performed to identify the predictors of attrition.

**Results:**

Based on individual patient data from the medical records, nearly 80% (95%CI: 76.7, 82.1) of the patients were retained in care in the first 3 and half years of antiretroviral therapy. After successfully tracing more than half of the LTFU patients, the updated one year retention in care estimate became 86% (95% CI: 83.41%, 88.17%). In the multivariate Cox regression analyses, severe immune deficiency at enrolment in care/or at cART initiation and ‘bed-ridden’ or ‘ambulatory’ functional status at the start of cART predicted attrition.

**Conclusion:**

Retention in HIV care in Addis Ababa is comparable with or even better than previous findings from other resource-limited as well as EU/USA settings. However, measures to detect and enroll patients in HIV care as early as possible are still necessary.

## Introduction

The advent of combination antiretroviral therapy (cART) has been accompanied by prolonged survival and improved quality of life among HIV infected persons worldwide, including resource-limited settings [1–5]. For instance, AIDS mortality has reduced by more than half within 5 years after cART became widely available in Addis Ababa, Ethiopia [6]. In addition, the number of new HIV infections is gradually decreasing as evidenced by a reduction in the national adult HIV incidence from 0.28% per year in 2004 to 0.03% per year in 2014 [7, 8].

However, poor retention in HIV care after patients started cART is recognized as a major challenge [9, 10]. Conceptually, retention in care and attrition complement each other, and current literature provides various, but closely related definitions [11–15]. The former generally refers to the proportion of cART taking patients known to be alive and still receiving medical care either on-site or at another site, including those stopping ARV medications (because of medical or personal reasons) while remaining in care. Attrition is defined as not being seen at the ART clinic for at least 1 month following patient’s most recent planned clinic visit or pharmacy refill appointment for reasons related to death or lost to follow-up (LTFU). Poor retention in care, particularly related to LTFU, can be devastating for patients’ lives, and also facilitates the occurrence of virologic failure, which in turn could lead to an increased chance of HIV transmission [16–19]

Previous studies about retention in care in Addis Ababa were conducted in the early years of roll-out of cART, when the program was not yet fully formed and there was limited experience to retain all the patients who initiated cART in HIV care [9, 13]. The present study is conducted to estimate the current retention in care and to identify predictors of attrition, which may help health care practitioners, program managers and policy makers to improve the management of HIV-infected people on cART.

## Method

### Setting and study participants

**Fig 1:**
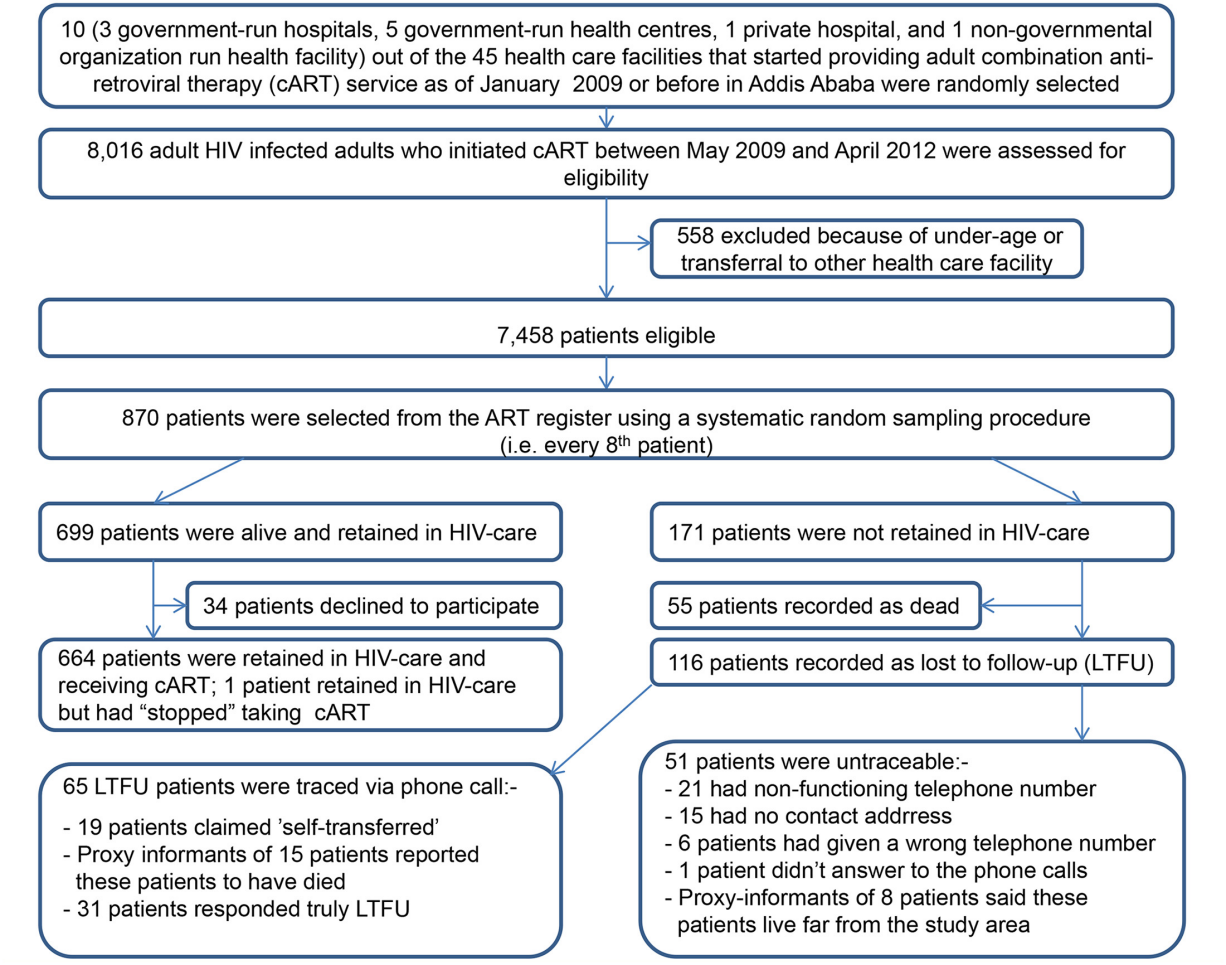
Flowchart depicting the random selection of health care facilities and patients included in the study.

This study was conducted between September 2012 and April 2013 in ten randomly selected health care facilities that had started providing adult cART service as of January 1, 2009 or before in Addis Ababa, Ethiopia. At the time of the study, HIV-infected adults who fulfilled the following World Health Organization (WHO) criteria were automatically eligible for cART once their readiness to initiate anti-retroviral therapy was assured: WHO clinical stage IV, irrespective of CD4 cell count; WHO clinical stage III with a CD4 cell count of ≤350/mm^3^; all WHO clinical stages with CD4 cell counts ≤200/mm^3^ [20]. Patients eligible to participate were selected in a two-step procedure using the national ART register as a sampling frame. First, we selected all adult HIV-infected persons without previous exposure to cART—except for the prevention of mother to child transmission—who initiated cART between May 1, 2009 and April 30, 2012. Patients who had been transferred-in or formally transferred-out after initiating cART were excluded. Second, out of these patients, we selected every 8^th^ patient from the ART register using a systematic random sampling technique. Fig 1—Flowchart depicting the random selection of health care facilities and patients included in the study—illustrates the selection process.

### Ethical consideration

Ethical approval was obtained from the Institutional Review Boards (IRB) at the College of Health Sciences, Addis Ababa University, and Millennium Medical College, St. Paul Hospital. Patients who were alive and still receiving medical care gave informed verbal consent. Verbal consent was preferred (over written consent) because a substantial proportion of the study participants were illiterate. Also, given the very sensitive nature of being HIV-positive in Ethiopia, obtaining a written consent (via signature or finger prints) could have potentially violated patients' privacy and anonymity. For patients who were not-retained in care, consent for tracing had already been obtained at start cART as part of the routine clinic follow-up and pharmacy refill procedures. Data were retrieved anonymously from medical registers using patients’ unique ART number or medical record number.

### Outcome measures

The current status in HIV care of each selected patient was determined based on the information in the ART register or patient follow-up card. It was typically recorded as died (of any cause), lost, dropped, stopped or retained in care. A patient defined as “lost” had missed his or her next clinic or pharmacy refill appointment for at least 1 month and at most 3 consecutive months. A patient defined as “dropped” had missed his or her planned clinic or pharmacy refill appointment for more than 3 consecutive months. Patients known to be alive and retained in care, but temporarily discontinued taking cART due to medical or personal reasons were defined as “stopped”. Patients were defined as ‘retained in care’ if they were alive and known to be still receiving medical care at the time of the study, including those considered as “stopped.” Attrition was defined as the number of patients documented as died, lost or dropped. Both patients who had been lost or dropped were considered as LTFU.

### Independent variables

Socio-demographic variables at enrolment in HIV care including age, sex, marital status, religion, educational level, and HIV-status disclosure were considered as potential predictors of attrition. The following variables were considered as additional predictors: dates of HIV diagnosis, enrolment in HIV care and cART initiation, type of antiretroviral treatment regimen, and evidence of tuberculosis at or after start cART. In addition, WHO clinical stage, functional status, CD4 cell count, and hemoglobin level were collected from the intake form or follow-up card. The following variables were also included in the analysis: time since start cART at study entry (‘date of the last known clinic or pharmacy refill visit’ minus ‘date of cART initiation’), and time since HIV diagnosis at start cART (‘date of cART initiation’ minus ‘date of HIV diagnosis’).

### Data extraction from the medical records

Patient data were extracted from the medical records using a Case Report Form (CRF) by data clerks. Contact addresses of all patients who had been lost or dropped were collected by the first author, which were later used for tracing purposes. Deaths already known by the ART clinic during the routine follow-up care were collected from the medical records. For patients who had died or become LTFU, dates of the last known clinic or pharmacy refill visit were recorded.

### Tracing of LTFU patients

In the participating facilities, it is a routine practice that patients who become LTFU are traced by adherence case managers and data clerks using telephone contacts.

In the present study, tracing of LTFU patients was carried out by the first author. First, all LTFU patients eligible for tracing were identified from the ART register. Next, patients’ or their caretakers’ telephone number together with information about whether the patient had disclosed his/her HIV status or not and to whom were extracted from the intake form.

Up to three attempts to contact LTFU patients by phone were made to ascertain their current status. When the patient himself/herself answered the call, he/she was first informed that the phone call was from the health facility where he/she initiated treatment, and he/she was kindly asked whether the timing was convenient. When the patient responded positively (otherwise an appointment was made for another round of call) he/she was asked the reasons for staying away, without mentioning anything about HIV/AIDS or cART until a confidentiality was established. In addition, they were asked if currently accessing any follow-up medical care, and when replied yes, they were requested to name the health facility for anti-retroviral drug refill, the re-start date, and whether they had discontinued cART in between; LTFU patients who named a legitimate ART site were generally re-classified as ‘retained in-care.’ For patients confirming not accessing any medical care, possible reasons for discontinuation of cART were explored further and recorded. In these cases, patients were advised to re-start medical care as quickly as possible. When a proxy informant (family member or friend) answered the phone call, s/he was kindly asked about the current address of the LTFU patient, and depending on the response, patient’s current vital status (alive or dead) was ascertained. LTFU patients who were reported as dead or confirmed to be truly LTFU were considered as “not-retained in care.” Patients who did not answer phone calls, those whose current vital status was ascertained via proxy informants, and those with no, non-functioning or a wrong telephone number were assumed to be “not-retained in care” in one analysis option, and “retained in care” in an alternative analysis. The status on chronic HIV care of all other patients was taken from the medical records.

### Statistical analyses

Retention in HIV care was the primary outcome of interest, and it was estimated using three different, but closely related analytic approaches. First, it was calculated as the number of patients known to be alive and receiving cART at the time of the study, including patients considered as “stopped,” divided by the total number of patients who started cART. This calculation was based on individual patient data routinely collected and available in the medical records. In the second approach, patients who were formally documented to be LTFU, but were successfully traced and claimed to be accessing medical care elsewhere with-out the knowledge of the facility where they had initiated cART (“self-transferred”) were re-classified as ‘retained in care’ and added to the numerator. In this approach, all untraceable LTFU patients were assumed “not-retained in care.” Analyses to identify predictors of attrition are based on estimates of this second approach. The third analytic approach was identical to the second approach, except that untraceable LTFU patients were assumed to be ‘retained in care’ and added to the numerator.

Time-to-attrition was the other end point of the study. It was calculated (in months) as the difference between the dates of cART initiation and the last known clinic or pharmacy refill visit. For all time-to-attrition analyses, time zero was set at the date of cART initiation. All-cause attrition was established at the recorded or reported date of death, last known date of clinic/or pharmacy refill visit, or final study date (April 30, 2013). Follow-up time for LTFU patients who were successfully traced and claimed to be “self-transferred” was censored at the date of phone call tracing.

Since the WHO immunological criteria (with/or without clinical staging) is commonly used for assessing eligibility to initiate cART in Addis Ababa [20], survival probabilities of retention in care stratified by this variable was estimated using the Kaplan-Meier method. The log-rank test was used to compare probability estimates of retention in care between the survival curves. Cox proportional hazards regression was performed to identify the variables that predicted post-cART attrition. The Hazard Ratio (HR) in the Cox regression model estimates the relative likelihood of attrition at consecutive points in time after cART initiation.

Predictor variables with a p-value of <0.2 were first identified in a univariate Cox regression analyses. Then, a multivariate Cox regression model was fitted by adding back in to the model, one at a time, variables not selected at a previous step. Likelihood ratio tests were used to compare between the fit of various models. Finally, variables that significantly (P-value <0.05) predicted attrition were kept in the final model. Proportional hazards assumption was checked by Schoenfeld-tests of residuals. EPI-data version 3.1 was used for data-entry and SPSS version 17 and STATA version 11 for statistical analyses.

## Results

### Patients

Characteristics of patients are summarized in Table 1. Of the total 870 eligible patients, 34 declined participation, and 836 patients were included in the present analyses. At enrolment in care, the median CD4 cell count was 141 (IQR, 73–239) cells/μL, and 203 (24.5%) patients were in WHO clinical stage I, 245 (29.6%) in stage II, 293 (35.3%) in stage III, and 88 (10.6%) in stage IV condition. In addition, 631 (79.6%) patients had a working functional status, 137 (17.3%) ambulatory, and 25 (3.2%) were bed-ridden.

**Table 1:** Patients’ socio-demographic, clinical, and treatment characteristics (n = 836).

Patient characteristic	Value*	
**Age—years** ^a^, **mean (SD) (n = 833)**	35	(8.9)
**Sex—male** ^a^	324	(38.8%)
**Educational status** ^a^, **(n = 795)**		
No education	112	(14.1%)
Primary	267	(33.6%)
Secondary	330	(41.5%)
Tertiary	86	(10.8%)
**Marital status** ^a^, **(n = 796)**		
Never married	186	(23.3%)
Married or cohabiting	381	(47.9%)
Divorced or separated	136	(17.1%)
Widowed	93	(11.7%)
**Religion** ^a^, **(n = 795)**		
Christian	741	(93.2%)
Muslim	51	(6.4%)
Other	3	(0.4%)
**Substance use/misuse—yes** ^a^, **(n = 686)**	144	(21.0%)
**Body Mass Index (kg/m^2^)** ^a^, **mean (SD) (n = 419)**	20.6	(3.38)
**Employment status** ^a^, **(n = 617)**		
Unemployed	199	(32.3%)
Not working/studying due to ill health	73	(11.8%)
Working full time	299	(48.5%)
Working part time	46	(7.5%)
**HIV status disclosure (to family/friend/neighbor)—yes** ^a^, **(n = 612)**	572	(93.5%)
**Functional status** ^b^, **(n = 817)**		
Working	677	(82.9%)
Ambulatory	113	(13.8%)
Bed ridden	27	(3.3%)
**WHO clinical stage** ^b^ ^,^ ^d^, **(n = 830)**		
Stage I	191	(23.0%)
Stage II	231	(27.9%)
Stage III	309	(37.2%)
Stage IV	99	(11.9%)
**First line drug regimen** ^b^		
TDF-containing	434	(51.9%)
AZT-containing	322	(38.5%)
d4T-containing	80	(9.6%)
**CD4 cells/μL** ^b^, **median (IQR) (n = 828)**	133.5	(72-to-203)
**Hemoglobin (g/dL)** ^b^, **mean (SD) (n = 709)**	13.1	(2.2)
**Evidence of tuberculosis at or after start cART—yes, (n = 817)**	151	(18.5%)
**Months since HIV diagnosis** ^b^, **median (IQR), (n = 822)**	1	(0-to-7)
**Months since start cART** ^c^, **mean (SD), (n = 836)**	22.6	(12.5)

* values are n (%) unless otherwise indicated.

^a^ at enrolment in HIV care.

^b^ at start cART.

^c^ at study entry or at last known clinic/pharmacy refill visit date.

^d^ according to the revised World Health Organization clinical staging of HIV/AIDS for adults and adolescents, 2005 [51].

TDF, Tenofovir; AZT, Zidovudine; d4T, Stavudine.

SD, standard deviation; IQR, inter-quartile range.

### Patients’ status in HIV care after cART initiation

As of April 30, 2013, 665 (79.5%; 95% CI: 76.7, 82.1) alive patients, including 1 ‘stopped’ case, were registered as retained in care, while 55 (6.6%), 11 (1.3%) and 105 (12.6%) were initially recorded as died, lost and dropped, respectively. Of the 116 LTFU patients, 65 (56%) were successfully traced: 31 were confirmed as truly LTFU, 19 claimed that they had been “self-transferred” after having had discontinued cART from 1 week to 22 months, and 15 patients were reported to have died of an unidentified, but illness-related cause, within 1 to 6 months of their last clinic/or pharmacy refill visit. The most frequently mentioned reasons for being LTFU included being away from home for various reasons (n = 12), searching for religious cure/holy water use (n = 6), lack of family support (n = 3), ‘no substantive reasons’ (n = 2), feeling healthy (n = 2), fear of health care providers’ reaction to patient’s return to HIV care (n = 1), fed-up with taking antiretroviral drugs (n = 1), fear of drug side effects (n = 1), and stigma/discrimination (n = 1). Two patients refused to provide further details. Of the remaining 51 “LTFU patients” whom we were unable to trace, 21 had non-functioning telephone numbers, 15 had no recorded contact address, 6 had given a wrong telephone number, and 1 patient did not answer the phone call. In addition, proxy informants of 8 LTFU-patients said that these patients were alive and currently living far from the study area.

### Estimating retention in care after cART initiation

Using individual patient data in the medical records, 79.5% (95% CI: 76.7, 82.1) were initially classified as retained in care by April 30, 2013. After successfully tracing 65 out of 116 LTFU patients, and assuming that the remaining 51 untraceable patients were ‘truly LTFU’, the updated retention in care estimate increased to 81.8% (95% CI: 79.1, 84.3) in the first 3 and half years of cART. With this estimate, the median time to attrition was 5 (IQR, 0 to 10) months while the median time between patients’ last known clinic visit and date of tracing was 16 (IQR, 8.3 to 26) months. The cumulative proportion of patients retained at 6, 12, 24, and 36 months after cART initiation was 90.67% (95% CI: 88.49%, 92.46%), 85.97% (95% CI: 83.41%, 88.17%), 82.70% (95% CI: 79.84%, 85.19%), and 79.04% (95% CI: 75.57%, 82.08%), respectively. With the alternative assumption that the 51 untraceable LTFU patients were “self-transferred”, the retention in care estimate further increased to 87.9% (95% CI: 85.5, 89.9) at the end of the current analysis.

### Retention in care versus CD4 cell count and functional status at start cART

**Fig 2:**
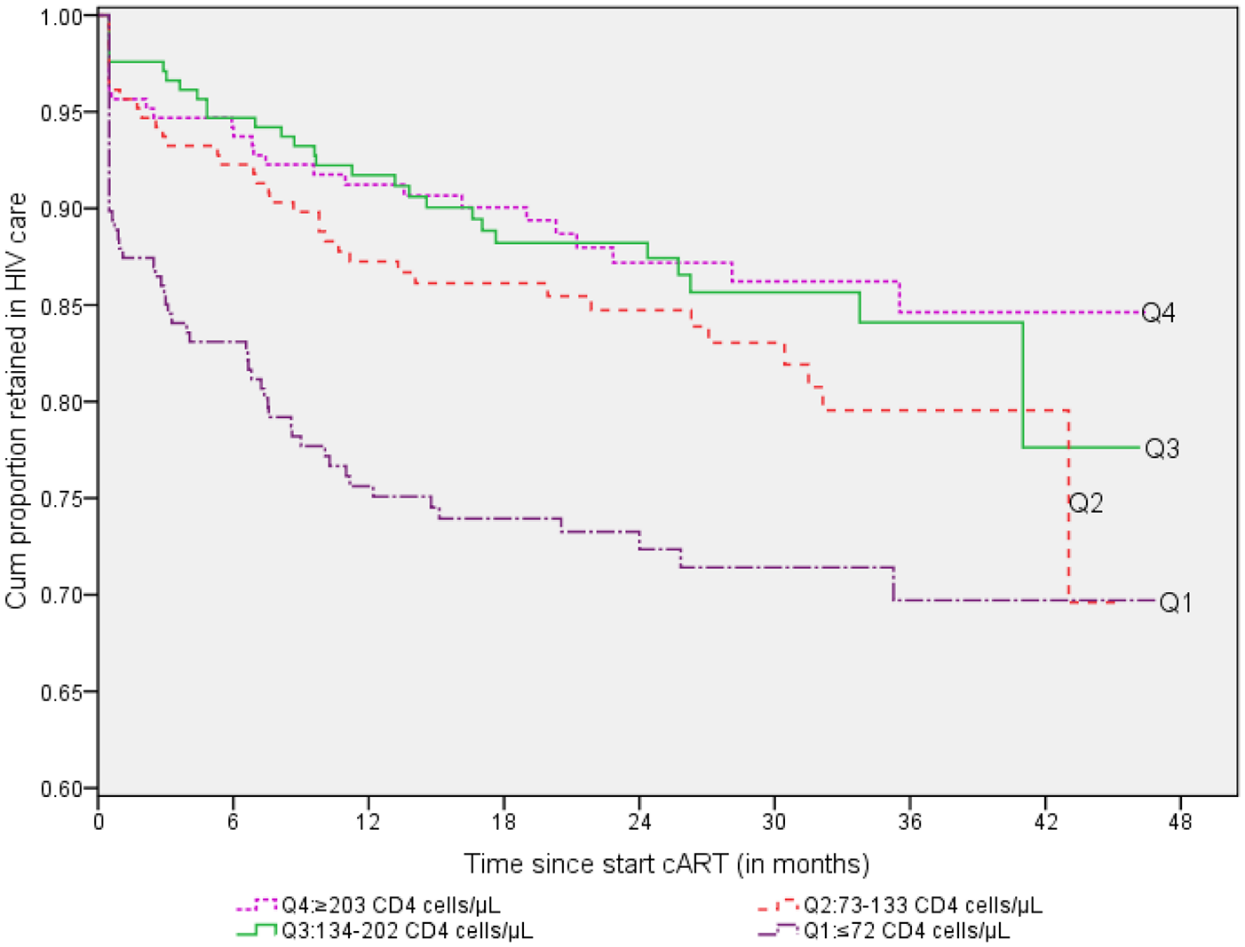
Kaplan-Meier survival curve of retention in HIV care by quartiles of CD4 cell count (cells/μL) at the start of cART, Addis Ababa, 2013 (n = 836).

**Fig 3:**
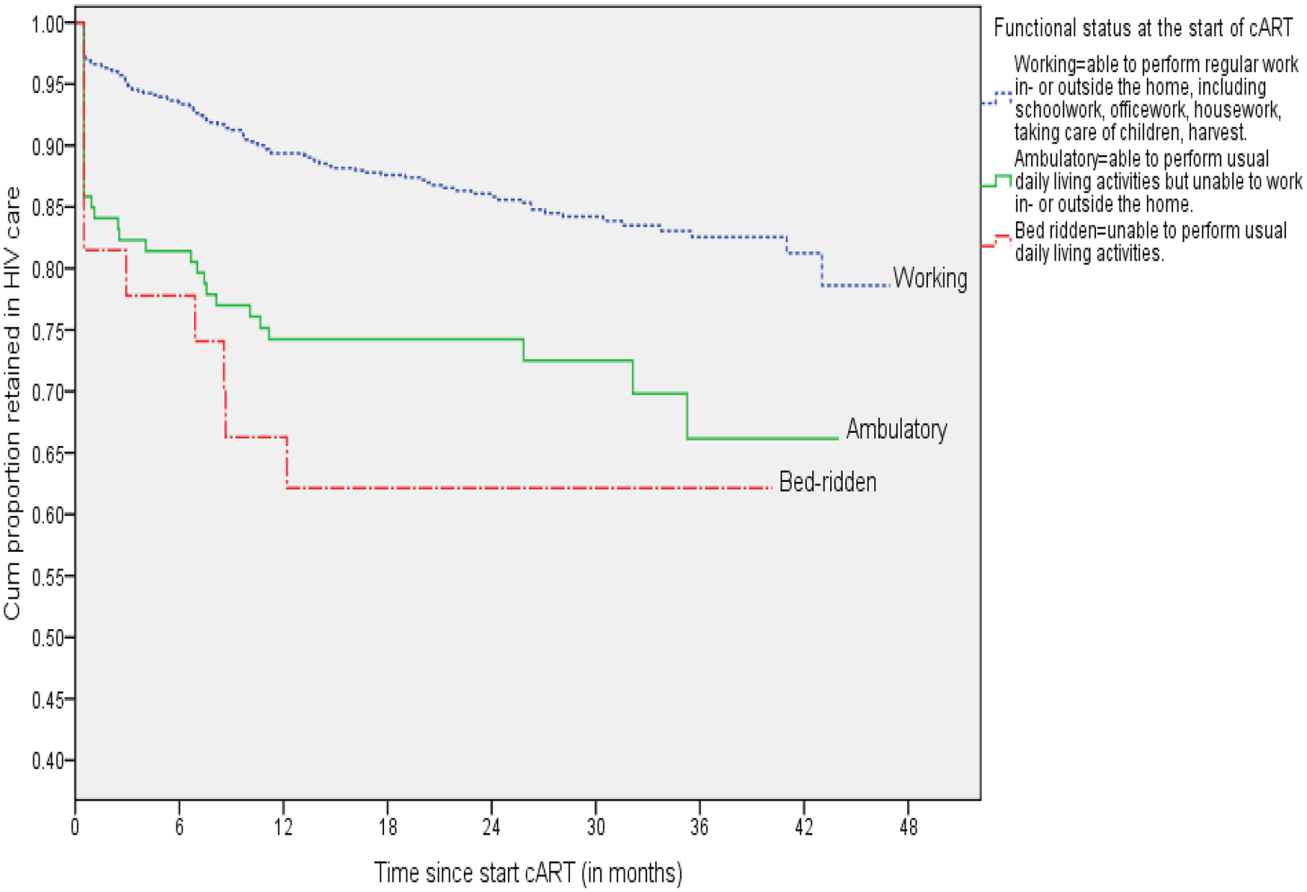
Kaplan-Meier survival curve for retention in HIV care by functional status at the start of cART, Addis Ababa, 2013 (n = 836).

Of the total 828 patients with a recorded CD4 cell count at the start of cART, 57 had died or become LTFU in quartile 1 (≤72 CD4 cells/μL), 36 in quartile 2 (73–133 CD4 cells/μL), 28 in quartile 3 (134–202 CD4 cells/μL), and 26 in quartile 4 (≥203 CD4 cells/μL). The Kaplan-Meier survival curve in Fig 2 –Kaplan-Meier survival curve of retention in HIV care by quartiles of CD4 cell count (cells/μL) at the start of cART—shows that patients who started cART in quartiles 4, 3, and 2 appeared more likely to be retained in care than patients in quartile 1 (log-rank Chi-sq statistic = 30.8, df = 3, p-value <0.001). In addition, patients with ‘bed-ridden’ or ‘ambulatory’ functional status at start cART had significantly lower probability of being retained in care than patients with a ‘working’ functional status (log-rank Chi-sq statistic = 24.53, df = 2, p-value <0.001; Fig 3 –Kaplan-Meier survival curve for retention in HIV care by functional status at the start of cART).

### Predictors of attrition from care

Results of the univariate and multivariate Cox regression analyses for predictors of attrition are presented in Table 2. The following variables assessed at enrolment in care had a substantial proportion of missing values and were therefore not entered in the multivariate analyses: employment status (n = 617), substance use/misuse (n = 686), body mass index (BMI) (n = 419), and hemoglobin level at start cART (n = 709).

**Table 2:** Results of the Cox regression model showing predictors of attrition from HIV care among HIV infected adults on cART.

Patient characteristic	Retained in HIV care(n = 684)	Not-retained in HIV care(n = 152)	Univariate analysis HR (95% CI)	Multivariate analysis HR (95% CI)
**Demographics**				
**Age** ^a^, **(n = 833)**				
18–24 yrs	42 (5.0%)	15 (1.8%)	1.58(0.93, 2.70)*	1.47(0.37, 1.08)
>24 yrs	639 (76.7%)	137 (16.4%)	1	1
**Sex** ^a^, **(n = 836)**				
Male	252 (30.1%)	72 (8.6%)	1.48(1.07, 2.03)*	1.32(0.94, 1.87)
Female	432 (51.7%)	80 (9.6%)	1	1
**Educational status** ^a^, **(n = 795)**				
Tertiary	74 (9.3%)	12 (1.5%)	0.77 (0.38, 1.58)	
Secondary	274 (34.5%)	56 (7.0%)	0.93 (0.56, 1.55)	
Primary	230 (28.9%)	37 (4.7%)	0.76 (0.44, 1.32)	
No education	92 (11.6%)	20 (2.5%)	1	
**Marital status** ^a^, **(n = 796)**				
Married or co-habiting	332 (41.7%)	49 (6.2%)	0.71 (0.46, 1.11)*	0.85(0.53, 1.36)
Divorced or separated	111 (13.9%)	25 (3.1%)	1.03 (0.61, 1.74)	1.23(0.69, 2.19)
Widowed	73 (9.2%)	20 (2.5%)	1.22 (0.69, 2.14)	1.51(0.80, 2.83)
Never married	154 (19.3%)	32 (4.0%)	1	1
**Characteristics at enrolment in HIV care**				
**HIV status disclosure (to family/closest friend/ neighbor)** ^a^, **(n = 836)**				
Yes	490 (58.6%)	82 (9.8%)	0.49 (0.35, 0.69)*	1.85(0.86, 3.95)
No	30 (3.6%)	10 (1.2%)	0.94 (0.48, 1.83)	0.83(0.54, 1.28)
Unknown	164 (19.6%)	60 (7.2%)	1	1
**CD4 count (cells/μL)** ^a^, **(n = 821)**				
Quartile1 (0–73)	148 (18.0%)	60 (7.3%)	3.33 (2.01, 5.53)*	3.03(1.76, 5.22)^‡^
Quartile2 (74–141)	170 (20.7%)	35 (4.3%)	1.77 (1.02, 3.06)	1.78(1.00, 3.16)^‡^
Quartile3 (142–239)	174 (21.2%)	30 (3.7%)	1.52 (0.87, 2.68)	1.51(0.83, 2.75)
Quartile4 (240–1,163)	184 (22.4%)	20 (2.4%)	1	1
**Characteristics at cART initiation**				
**Functional status** ^b^, **(n = 817)**				
Bed ridden	17 (2.1%)	10 (1.2%)	3.09(1.61, 5.93)*	2.22(1.10, 4.46) ^‡^
Ambulatory	81(9.9%)	32 (3.9%)	2.13(1.43, 3.17)	1.68(1.10, 2.58) ^‡^
Working	576(70.5%)	101 (12.4%)	1	1
**WHO clinical stage** ^b^ ^,^ ^c^, **(n = 830)**				
Stage IV	74 (8.9%)	25 (3.0%)	1.93(1.11, 3.34)*	0.61(0.33, 1.16)
Stage III	241 (29.0%)	68 (8.2%)	1.65(1.05, 2.59)	1.05(0.62, 1.78)
Stage II	201 (24.2%)	30 (3.6%)	0.92(0.54, 1.55)	0.88(0.44, 1.78)
Stage I	165 (19.9%)	26 (3.1%)	1	1
**Evidence of tuberculosis at or after start cART, (n = 817)**				
Yes	120 (14.7%)	31 (3.8%)	1.24 (0.84, 1.85)	
No	552 (67.6%)	114 (14.0%)	1	
**First line drug regimen** ^b^, **(n = 836)**				
d4T-containing	60 (7.2%)	20 (2.4%)	1.11 (0.68, 1.82)*	1.12(0.63, 2.02)
AZT-containing	275 (32.9%)	47 (5.6%)	0.64 (0.45, 0.92)	0.88(0.58, 1.34)
TDF-containing	349 (41.7%)	85 (10.2%)	1	1
**Months since HIV diagnosis** ^b^, **(n = 822)**				
≤1 month	377 (45.9%)	94 (11.4%)	1.29 (0.92, 1.79)	
>1 month	295 (35.9%)	56 (6.8%)	1	

^a^ at enrolment in HIV care.

^b^ at start cART.

^c^ according to the revised World Health Organization clinical staging of HIV/AIDS for adults and adolescents, 2005 [51].

d4T, Stavudine; AZT, Zidovudine; TDF, Tenofovir.

HR, hazard ratio.

* p-value < 0.2.

^‡^ p-value < 0.05.

1 = reference category.

Based on the 81.8% retention in care estimate, CD4 cell count at enrolment in care (quartile1 vs. quartile4: HR = 3.03, 95%CI: 1.76, 5.22; quartile2 vs. quartile4: HR = 1.78, 95%CI: 1.00, 3.16; quartile3 vs. quartile4: HR = 1.51, 95%CI: 0.83, 2.75)/or at cART initiation (quartile1 vs. quartile4: HR = 2.18, 95%CI: 1.33, 3.57; quartile2 vs. quartile4: HR = 1.41, 95%CI: 0.84, 2.38; quartile3 vs. quartile4: HR = 1.04, 95%CI: 0.59, 1.82) and functional status at start cART (bed-ridden vs. working: HR = 2.22, 95%CI: 1.10, 4.46; ambulatory vs. working: HR = 1.68, 95%CI: 1.10, 2.58) remained independently predictive of attrition in the multivariate model. These variables remained also significantly associated with attrition based on the alternative retention in care estimates of 79.5% and 87.9%. Moreover, men were more likely to experience attrition compared to women based on the 79.5% estimate (HR = 1.42; 95%CI: 1.02, 1.99).

## Discussion

In this large multi-center study, the current retention in care estimate of 86% in the first 12 months of cART is in line with [21–23] or better than [12, 24–27] previous study findings from other resource-limited settings. Similarly, retention in care was comparable with or even higher than estimates from Europe or the USA [28–32] although strict comparison may not be appropriate as studies in these settings measured retention along the whole cascade of care from HIV testing to viral load suppression [33–35]. However, our result is lower than the one year nation-wide retention in care estimate of 92% in a recently published article from Ethiopia [36]. The most likely explanation is the continuous influx and referral of severely immuno-compromised patients to the health facilities in Addis Ababa for better treatment, who may have died shortly after the start of cART, or simply returned back to their place of origin without a formal transfer-out procedure.

The higher risk of attrition in the first 6 months of antiretroviral therapy, a period considered crucial for the long-term success of cART [37], is in agreement with previous reports from Ethiopia [9, 10, 13] and other African countries [25, 37–39]. Notably, of the total number of patients who were not retained in care, nearly half had died and the remaining 54% had become LTFU. Previous studies in Africa have reported that AIDS-related causes including mycobacterial and neurologic infections, malnutrition/anemia, drug toxicity, immune-reconstitution inflammatory syndrome (IRIS), and septicemia may be the most likely causes [22, 40–42]. In addition, a considerable number of the LTFU patients who were traced and directly interviewed pointed out that they had run out of ARV medications while away from home for different reasons. Therefore, the current practice of antiretroviral drug prescription for 1 to 3 months in the routine follow-up care, or for 1 month as an emergency [20], needs to be relaxed further to accommodate individual patient needs and special circumstances.

A sizable proportion of the LTFU patients who claimed to be “self-transferred” reported that they re-started taking cART in the new facility as if they were naive. These kinds of practices have been reported previously [9, 43], and may have implications on ART program outcome evaluations and resource allocation at the regional and/or national level [44]. An additional issue to consider is the substantial proportion of LTFU patients whom we were unable to trace because of reasons mainly related to incomplete, non-functioning or no recorded contact address. Measures to strengthen the recording of accurate information with alternative contact addresses at the enrolment of patients in HIV care are required at the facility level.

An interesting finding is that two-thirds of the HIV-infected patients had already been in a state of advanced immune deficiency (i.e. <200 CD4 cells/μL) by the time they were enrolled in HIV care. Severe immune deficiency of ≤73 CD4 cells/μL at enrolment in care was found to be the strongest predictor of attrition and adds to evidence of several other studies [25, 37, 44, 45]. As a strategy, the WHO and national treatment guidelines have been continuously updated to raise the CD4 cell count threshold of initiating cART well above 200 cells/μL [20, 46, 47]. However, this initiative should be supported by identification and enrollment of patients in care as early as possible [48, 49]. In addition, the causes of late enrolment in care with advanced immunodeficiency in a setting where HIV counseling and testing is widely available, and knowledge of where to get a HIV test is nearly 100% [50], should be further scrutinized and addressed.

This study has important strengths. The larger sample size coupled with the random selection of study facilities and patients facilitate generalization of the findings to adult patients from urban areas. Moreover, meticulous ascertainment of LTFU patients’ current status in HIV care via phone call tracing helped to better estimate the level of retention in care and understand some of the reasons for attrition from the patients’ perspective, e.g., patients had run out of ARV medications while away from home.

Results from this study should be interpreted cautiously. First, contact addresses for nearly half of the LTFU patients were either missing or non-functioning. This might have underestimated the true level of retention in care. Conversely, retention might also have been overestimated because attrition (due to LTFU) was ascertained only once during the study period. Since LTFU may occur more than once in the course of treatment for an individual patient, patients classified as “retained in-care” may have previously been LTFU. Future studies may need to prospectively ascertain patients’ status across the continuum of care for a better and more refined estimate. Secondly, it is possible that LTFU patients’ responses might be affected by perceived social desirability. Thirdly, the follow-up period in the present study is relatively short and the results may not be applicable to longer follow-up periods. Finally, confounding due to unmeasured characteristics can’t be ruled out.

In summary, the current retention in care estimate among HIV-infected adults receiving cART in Addis Ababa is high and comparable with or even better than several previous study results from resource-limited and resource-rich settings. However, early attrition from care shortly after cART initiation is still a problem. Severe immune deficiency at enrolment in care/or at cART initiation and poor functional status at start cART were important predictors of attrition. Therefore, earlier identification of HIV-infected people and enrolling them in care as early as possible should be part of future interventions.
